# Properties and ecological assembly mechanisms of microbial communities across salinity levels in coastal saline-alkaline soils

**DOI:** 10.1186/s12870-026-08391-3

**Published:** 2026-03-20

**Authors:** Chao Ji, Kun Yan, Jinyuan Zhai, Lexin Sun, Jingrun Wang, Mengliu Shen, Meiying Liu, Qitong Wang

**Affiliations:** 1 College of Advanced Agriculture and Life Sciences, Weifang University, Weifang, 261061 China; 2 College of Forestry, Shandong Agriculture University, Tai’an, 271018 China

**Keywords:** Yellow River Delta, Saline-alkaline land, Microbial community, Soil salinization, Microbial functional attributes

## Abstract

Microorganisms play key roles in nutrient cycling and soil remediation in saline-alkaline ecosystems. However, how their assembly rules and interaction networks change during natural succession remains unclear. We used a space-for-time substitution approach to investigate soil microbial communities along a salinity chronosequence in the Yellow River Delta, covering three successional stages: high salinity (HS), medium salinity (MS), and low salinity (LS). We performed 16S rRNA and ITS amplicon sequencing on 24 composite soil samples and analyzed community composition, functional traits, assembly processes, and biotic association networks. The results showed that, environmental filtering—especially by soil organic carbon (SOC) and electrical conductivity (EC)—was the primary driver of community structure. Functionally, low-salinity soils were enriched in nitrogen-cycling genera (e.g., *Nitrospira*) and arbuscular mycorrhizal fungi, whereas high-salinity soils favored saprotrophs and sulfur-metabolizing taxa. Importantly, null-model analysis revealed a fundamental shift in assembly mechanisms. In harsh high-salinity habitats, stochastic processes dominated community assembly (normalized stochasticity ratio, NST > 0.5), and bacterial communities formed highly positive, cooperative networks. By contrast, as salinity decreased, deterministic processes prevailed (NST < 0.5). Meanwhile, negative edges increased significantly (*P* < 0.05), suggesting that relaxed environmental stress intensified interspecific competition and exclusion. Cross-domain network analysis further indicated that under high-salinity conditions, bacteria and fungi adopted a more tightly coordinated survival strategy. These findings suggest that restoration of saline-alkaline land should not only reduce salinity, but also steer the balance between deterministic selection and stochastic colonization to build stable, multifunctional microbial networks. This provides a theoretical basis for the targeted development of salt-tolerant microbial inoculants and for optimizing vegetation restoration schemes.

**Supplementary Information:**

The online version contains supplementary material available at 10.1186/s12870-026-08391-3.

## Introduction

Global climate change has increased the frequency of extreme weather events. Together with intense human activities, it has made soil salinization one of the most common drivers of land degradation in arid, semi-arid, and coastal regions worldwide [20, 64, 68, 69]. Salinity is a major abiotic stress for plants and can cause severe losses in crop yield and quality through osmotic stress and ion toxicity [5, 6]. This process not only reduces soil fertility, but also markedly alters carbon and nitrogen cycling, thereby affecting microbial activity and ecosystem functioning. In natural ecosystems, soil microorganisms are highly sensitive to environmental change and can respond rapidly [57]. These responses include shifts in microbial diversity, community structure, and functional traits. They are crucial for regulating nutrient cycling and maintaining soil health under stress. Therefore, microbial community structure and diversity are widely regarded as reliable indicators of soil nutrient quality across ecosystems [49].

Current research on soil microorganisms in coastal saline-alkaline environments mainly focuses on microbial diversity, community structure, and their static correlations with soil physicochemical properties at regional and global scales [74]. Many studies have documented spatial variation in soil microbial communities, providing detailed insights into links between biogeochemical processes and environmental change [23]. Despite these advances, we still know little about how long-term successional processes reshape microbial functional networks in these ecosystems. In particular, during natural recovery, how do the drivers of community assembly shift from salinity-dominated environmental filtering to processes dominated by biotic interactions? In addition, how vegetation succession regulates this assembly process by altering the soil organic carbon (SOC) pool remains a key unanswered question [27].

The Yellow River Delta is a continuously expanding alluvial plain formed by sediment deposition at the river estuary and is an important reserve of arable land in China. Located at the interface between terrestrial and marine ecosystems, this region exhibits a clear soil salinity gradient. As distance from the coastline increases, soil salinity decreases and soil properties change. Over the past four decades, natural wetland evolution within the delta (without human disturbance) has been dominant, with a landscape transfer dominance index (LTDI) of 52.74% [8]. This unique setting makes the delta an ideal “natural laboratory” for examining how environmental gradients shape microbial community dynamics [70]. Importantly, the region enables chronosequence research using space-for-time substitution, spanning coastal high-salinity bare flats and *Suaeda glauca* Bunge communities (early succession) to moderately salinized *Tamarix chinensis* Lour shrublands (mid-succession) [8]. Low-salinity *Robinia pseudoacacia* forests have been free from human disturbance for nearly 20 years and represent typical plantations in the Yellow River Delta [75]. The coupled evolution of vegetation and soil environments provides an excellent model for disentangling the relative contributions of salt stress and vegetation-driven biotic effects to microbial community assembly [16].

To better understand the filtering effects of salinity levels on soil microorganisms and the underlying assembly mechanisms, we selected three sites representing different successional stages: high salinity (HS), medium salinity (MS), and low salinity (LS). We comprehensively assessed soil physicochemical properties and microbial community structure across these across these salinity levels. This study addressed two key questions. (1) How do microbial community composition and functional features differ among soils with different levels of salinity? (2) What are the patterns and mechanisms of microbial interaction networks across soils with differing salt levels? During succession (i.e., the transition from high to low salinity levels), which processes play a more crucial role in microbial community assembly: deterministic processes or stochastic processes? By answering these questions, we aim to reveal ecological patterns of microbial community recovery in saline-alkaline ecosystems and provide a scientific basis for sustainable management and ecological restoration of saline-alkaline land.

## Materials and methods

### Study site and soil sampling

**Fig. 1:**
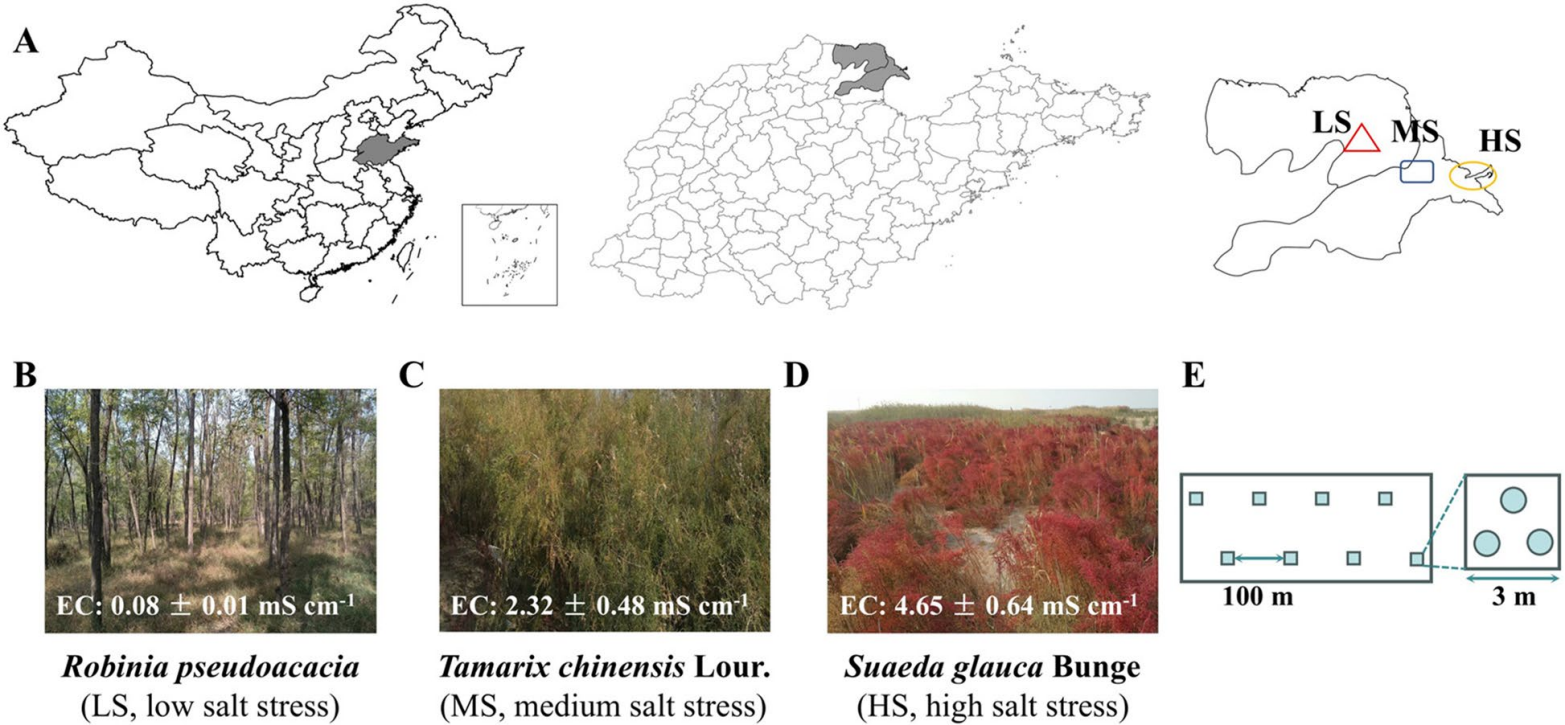
Geographical location of sampling points. **B** HS: Yellow ellipse marked area; (**C**) MS: Blue square labeled area; (**D**) LS: Area marked by a red triangle; (**E**) Sampling scheme: eight points per site (~ 100 m apart) in the sampling area (left), and three subsamples (3 m × 3 m) pooled into one sequencing sample (right)

The study area is located in the core protected zone of the Yellow River Delta. Its evolution can be divided into four stages: rapid deposition (1976–1980), channel widening (1980–1985), channel shrinkage (1985–1996), and channel incision and deepening (1996–2015) [77]. The study area is located within a temperate continental monsoon climate zone, with an average annual temperature ranging from 11.7 to 12.8 °C and an annual precipitation between 530 and 630 mm. Precipitation is predominantly concentrated in the summer months, with July and August accounting for 70% of the total annual rainfall. The average annual evaporation is approximately 1962 mm, resulting in an average evaporation-to-precipitation ratio of 3.22. Soil salinity in this region gradually decreases with increasing distance from the coastline. Based on significant differences in soil salinity [24], three sampling sites were selected: high salinity (HS, EC > 4 mS/cm, 119°10′31″E, 37°46′00″N), medium salinity (MS, 2 < EC < 4 mS/cm, 118°59′15″E, 37°45′45″N), and low salinity (LS, EC < 2 mS/cm, 118°46′47″E, 37°49′46″N) (Fig. 1A, B, C, D). The dominant vegetation at these sites is successively *Suaeda glauca* Bunge, *Tamarix chinensis* Lour., and *Robinia pseudoacacia*, respectively. Because soils in microbial ecology studies are often highly spatially heterogeneous, especially in saline-alkaline ecosystems where salinity, moisture, and organic matter are often patchily distributed. If sampling relies on a single point, individual samples can be strongly affected by microenvironments (e.g., salt crystallization or plant residues). This can inflate variance among samples and mask true differences among treatments. Therefore, we used a composite sampling strategy. Eight sampling points were established at each site, spaced approximately 100 m apart. Soil samples from each point consisted of a composite of three sub-samples collected from a 9 m^2^ area (Fig. 1E). All three sites have remained free from anthropogenic disturbance for nearly two decades (HS and MS have been undisturbed since their formation). Soil samples were collected from areas without plant roots, after removing the top 3–5 cm of surface soil and any visible plant or animal debris. Soil cores were taken from the 0–20 cm depth. The samples were stored at 4 °C and promptly transported to the laboratory for further processing. Each soil sample was evenly divided into two portions: one stored at −20 °C for DNA extraction and microbial diversity analysis, and the other air-dried for measuring basic soil physicochemical properties and amino sugar content.

### Determination of soil physicochemical properties

Total nitrogen (TN) was measured following the method described by [2]. Exchangeable potassium (K) was determined according to the procedure outlined by [56]. Soil organic carbon (SOC) content was analyzed using the potassium dichromate oxidation method [66]. Soil pH and electrical conductivity (EC) were measured in 1:2.5 and 1:5 soil–water ratios, respectively, using digital pH (FE20) and EC (FE930) meters (Mettler Toledo, Switzerland). Soil bulk density (BD) was calculated by the gravimetric method [65]. For ion analysis, 0.2 g of soil was treated with 1 mL deionized water and 5 mL concentrated sulfuric acid overnight; the resulting solution was diluted to 50 mL with deionized water. Measurements were conducted on 1 mL of the solution, further diluted 10-fold. The concentrations of Na^+^, K^+^, Ca^2+^, and Mg^2+^ were measured using inductively coupled plasma optical emission spectroscopy (ICP-OES, Thermo Scientific™ iCAP™ 7000 Plus, USA) [53].

### Microbial diversity and community structure analysis

Total DNA was extracted from soil samples using the FastDNA SPIN Kit for Soil (MP Biomedicals, Solon, OH, USA). Extracted genomic DNA served as the template for conventional PCR amplification. For bacteria, the V3–V4 region of the 16S rRNA gene was amplified with universal primers 338 F (5′-ACTCCTACGGGAGGCAGCA-3′) and 806R (5′-GGACTACHVGGGTWTCTAAT‐3′), while fungal ITS genes were amplified with primers ITS1F (5′- CTTGGTCATTTAGAGGAAGTAA-3′) and 2043R (5′-GCTGCGTTCTTCATCGATGC-3′). The PCR reaction mixture included 4 µL of 5 × FastPfu Buffer, 2 µL of 2.5 mM dNTPs, 0.8 µL of each primer (5 µM), 0.4 µL of FastPfu Polymerase, 10 ng of template DNA, and ddH_2_O to a final volume of 20 µL.

The PCR program for amplifying the bacterial V3–V4 region included an initial denaturation at 95 °C for 2 min, followed by 40 cycles of denaturation at 95 °C for 30 s, annealing at 55 °C for 30 s, and extension at 72 °C for 30 s, with a final extension at 72 °C for 5 min. For fungal ITS sequences, the PCR program began with an initial denaturation at 95 °C for 3 min, followed by 40 cycles of denaturation at 95 °C for 30 s, annealing at 55 °C for 30 s, and extension at 72 °C for 45 s, with a final extension at 72 °C for 10 min. Each sample was amplified in triplicate, and PCR products were pooled and visualized by 2% agarose gel electrophoresis.

Target PCR products were excised from the gel and purified using the AxyPrep DNA Gel Extraction Kit (AXYGEN) with Tris–HCl elution. Purified products were quantified with the QuantiFluor™-ST blue fluorescence quantitation system (Promega), and the MiSeq library was prepared accordingly. Paired-end sequences generated by MiSeq sequencing were processed by merging reads based on overlap, incorporating quality control filtering for read quality and merging efficiency. Valid sequences were identified by differentiating samples using barcode and primer sequences at both ends, with sequence orientation corrected to produce optimized data. The raw reads were deposited in the NCBI Sequence Read Archive database (accession number: PRJNA1206298 and PRJNA1206731).

Functional Prediction: (1) Functional predictions of bacterial 16S rRNA data were conducted using the FAPROTAX database, with the latest version used to mitigate the impact of 16S marker gene copy number variation in bacterial genomes [35]. (2) Fungal communities were classified into guilds based on published literature and authoritative online sources (FUNGuild, http://www.funguild.org/) [45]. (3) Pearson correlation analysis in SPSS software was used to evaluate the relationships between environmental factors and community functions. Environmental variables—including pH, electrical conductivity (EC), organic carbon, and total nitrogen—were correlated with the relative abundances of bacterial functional categories and fungal guild classifications across sampling sites. This analysis enabled summarization of the influence patterns of environmental factors on community functions.

### Habitat niche breadth, neutral model, and null model

Sampling sites along the salinity gradient (HS/MS/LS) were treated as discrete resource states. Levins’ niche breadth was used as a proxy for “habitat specialization” to quantify how evenly taxa were distributed along the salinity gradient. Levins’ niche breadth values for species were calculated using the “spaa” package in R v.4.0.2 (https://www.r-project.org/), and the average niche breadth across all species in a community was used to estimate habitat niche breadth [80]. The neutral model developed by [55] was employed to evaluate the role of neutral processes in community assembly. This model describes the relationship between species abundance and frequency, predicting a neutral-based assembly process within the model and suggesting a niche-based process when observations fall outside the model. In this model, *m* represents the species migration rate, with a higher *m* value indicating a lower dispersal limitation. A null model was applied using the “NST” R package to estimate the relative importance of stochastic and deterministic processes in community assembly. A null distribution of expected beta-diversity, based on Bray–Curtis distance, was generated by randomly shuffling the original communities, followed by calculation of the normalized stochasticity ratio (NST). NST was calculated separately within each habitat group (HS/MS/LS) to quantify the relative contributions of stochastic versus deterministic processes to community assembly within each salinity interval. An NST value below 0.5 indicates that deterministic processes dominate, while an NST value above 0.5 suggests dominance by stochastic processes [46].

### Network analysis and keystone species

This network analysis was conducted based on Spearman's rank correlation, and the *P*-values of the correlation matrix were corrected using the Benjamini-Hochberg (BH) method. Species with relative abundances exceeding 0.001 and present in at least 80% of samples were selected for analysis. Correlations with coefficients below 0.6 or *P*-values above 0.05 were excluded to ensure that only strong associations were retained. To enable the microbial co-occurrence network to more truly reflect the biological activities of the microbial community, we used iDIRECT and QCMI algorithms to filter the co-occurrence network and constructed the microbial biotic association network [33, 34, 72]. First, the iDIRECT package in Python (v3.8) was used to resolve spurious correlations caused by indirect environmental mediation, self-circulation, and interaction strength overflow. Second, the QCMI algorithm in R (v4.0.5) was employed to remove correlations induced by diffusion constraints and environmental filtering. This combined strategy segregates biotic from abiotic signals, ensuring that the final network correlations primarily represent microbial biological activities. Gephi v.0.9.2 was used to generate network diagrams and analyze topological parameters. In the network, nodes represent OTUs, and links between nodes indicate close relationships. A group of nodes that are highly connected within but less connected to outside nodes is referred to as a module.

### Statistical analyses

Data analysis was conducted using IBM SPSS 19.0. Soil parameters were normally distributed. One-way ANOVA was applied to compare differences among groups (*P* < 0.05). Redundancy analysis (RDA) and canonical correlation analysis (CCA) were performed using Canoco 4.5.1 (Microcomputer Power, Ithaca, USA) to examine the relationships between the relative abundances of bacterial and fungal taxa and soil chemical properties. The LEfSe tool’s non-parametric factorial Kruskal–Wallis sum-rank test was used to identify groups with significant differences in abundance. LEfSe employs linear discriminant analysis (LDA) to estimate the effect of each component’s (genus’s) abundance on group differences.

## Results

### Soil physicochemical properties

Significant differences were observed in the physicochemical properties of soils across the sites. All soils were strongly alkaline, with pH values exceeding 8.2. The HS site, positioned perpendicular to the coastline, had significantly higher pH, EC, and concentrations of Na, K, Ca, and Mg compared to the MS and LS sites (*P* < 0.05). In contrast, the LS site showed significantly higher bulk density, organic carbon, and total nitrogen levels than the MS and HS sites (*P* < 0.05) (Table 1).

**Table 1:** Physical and chemical properties of soil

Site	pH	Water %	EC mS/cm	BD g/cm^3^	SOC g/kg	TN g/kg	Na^+^ g/kg	K^+^ g/kg	Ca^2+^ g/kg	Mg^2+^ g/kg
HS	8.57 (0.07)^a^	12.41 (0.31)^a^	4.65 (0.64)^a^	1.56 (0.10)^b^	3.95 (1.14)^b^	0.21 (0.06)^b^	3.84 (0.65)^a^	0.14 (0.04)^b^	0.35 (0.08)^a^	0.28 (0.07)^a^
MS	8.47 (0.07)^b^	12.28 (0.32)^a^	2.32 (0.48)^b^	1.62 (0.16)^b^	2.81 (1.20)^b^	0.27 (0.04)^b^	2.12 (0.55)^b^	0.17 (0.04)^a^	0.28 (0.05)^b^	0.18 (0.04)^b^
LS	8.26 (0.08)^c^	7.28 (0.41)^b^	0.08 (0.00)^c^	1.75 (0.04)^a^	17.41 (2.44)^a^	0.68 (0.18)^a^	0.18 (0.05)^c^	0.03 (0.01)^c^	0.04 (0.01)^c^	0.02 (0.01)^c^

The data in the table are mean (standard error) of 8 replicates. Different lowercase letters a, b, c represent significant differences

### Soil microbial richness and diversity across different sites

Bacterial richness and diversity analyses showed that the Chao1 index was significantly higher at the HS and MS sites than at the LS site (*P* = 0.002), with mean values of 3979.90 (HS), 3635.20 (MS), and 3310.00 (LS); no significant difference was detected between HS and MS (Supplementary Fig. 2; Supplementary Table 1). For the Shannon index, bacterial diversity at HS and LS was significantly higher than that at MS (*P* = 0.001), with mean values of 6.51 (HS), 6.22 (MS), and 6.57 (LS); HS and LS did not differ significantly (Supplementary Fig. 2; Supplementary Table 1). In contrast to bacteria, fungal richness (Chao1) was significantly higher at the LS site than at the HS and MS sites (*P* < 0.001), with mean values of 105.23 (HS), 141.83 (MS), and 285.74 (LS) (Supplementary Fig. 2; Supplementary Table 1). However, fungal diversity (Shannon) did not differ significantly among the three sites, with mean values of 2.46 (HS), 2.86 (MS), and 2.64 (LS) (Supplementary Fig. 2; Supplementary Table 1).

### Diversity and composition of microbial communities in saline-alkaline soils

Across the three sites, 810 bacterial OTUs and 69 fungal OTUs were shared. Additionally, each site contained unique OTUs: HS exhibited 2,741 bacterial and 225 fungal OTUs, MS had 1,389 bacterial and 366 fungal OTUs, and LS contained 2,526 bacterial and 602 fungal OTUs (Supplementary Fig. 3).

**Fig. 2:**
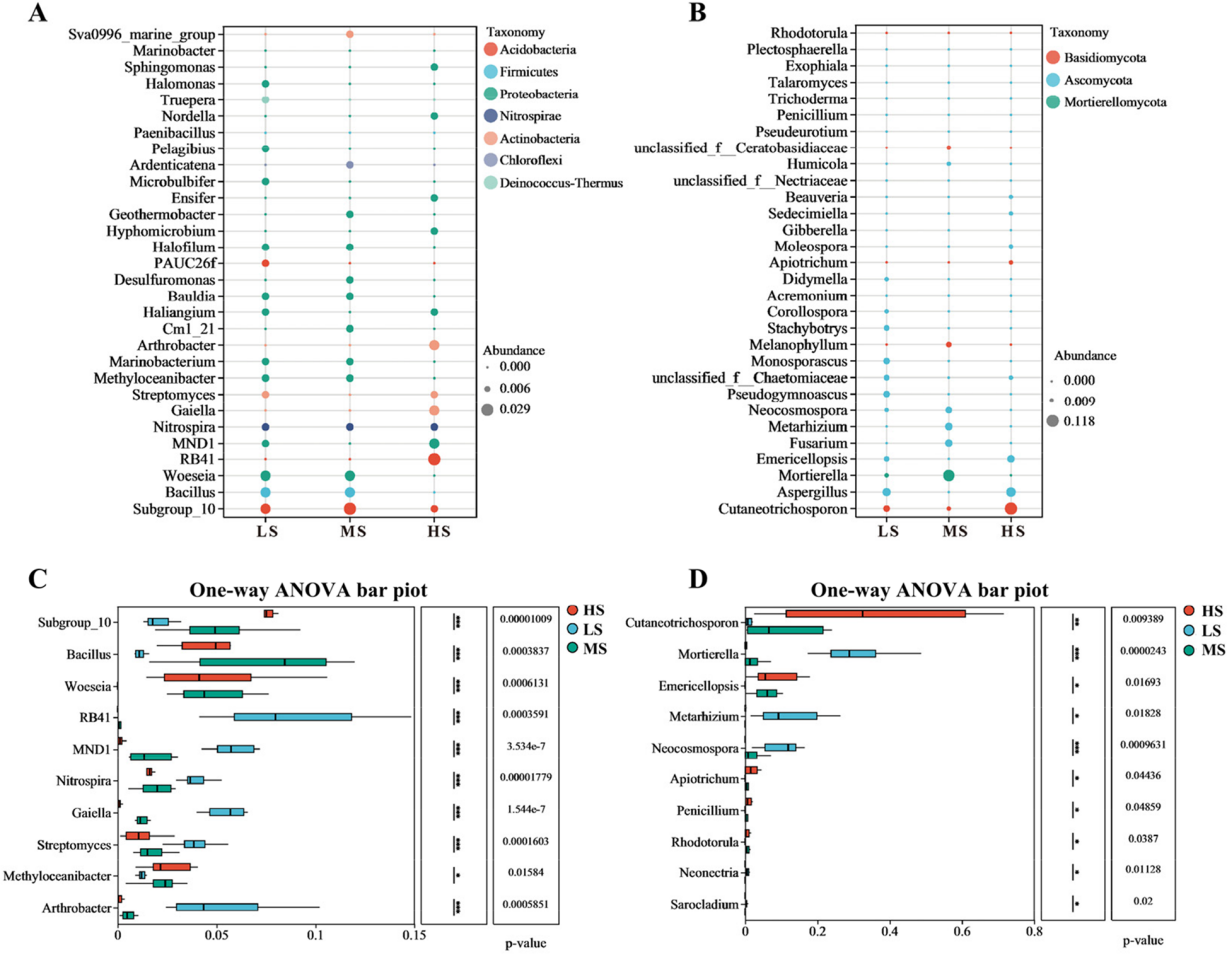
Significant differences in the relative abundance of bacterial and fungal genera across sampling sites. **A** Genus-level composition and relative abundance of bacteria across sampling sites. **B** Genus-level composition and relative abundance of fungi across sampling sites. **C** Statistical analysis of significant differences in bacterial genera across sampling sites. **D** Statistical analysis of significant differences in fungal genera across sampling sites. The y-axis represents genus-level names, and the x-axis indicates the average relative abundance of each species across groups. The far-right column displays *P* values, with significance levels denoted as follows: * 0.01 < *P* ≤ 0.05, ** 0.001 < *P* ≤ 0.01, *** *P* ≤ 0.001

Proteobacteria was the dominant phylum across all samples; however, significant differences in bacterial community composition were observed at the genus level (Fig. 2A, Supplementary Fig. 4 A, 5 A). In MS soils, the dominant genera were *Halomonas*, *Pelagibius*, and *Microbulbifer*. In HS soils, *Geothermobacter*, *Desulfuromonas*, and *Cm1_21* were predominant. In LS soils, *Sphingomonas*, *Nordella*, *Ensifer*, and *Hyphomicrobium* were the dominant genera.

Fungal communities at all sampling sites were dominated by the phylum Ascomycota, though distinct differences were observed at the genus level (Fig. 2B, Supplementary Fig. 4B, 5B). In MS soils, the dominant fungal genera were *Didymella*, *Corollospora*, *Stachybotrys*, *Monosporascus*, *Pseudogymnoascus*, and *Neocosmospora*. In HS soils, the predominant genera were *Beauveria*, *Sedecimiella*, and *Moleospora*. In LS soils, the dominant fungal genera included *Humicola*, *Metarhizium*, and *Fusarium*.

One-way ANOVA was conducted to analyze significant differences in dominant bacterial genera across sampling sites (Fig. 2C). The results showed that *Subgroup_10*, *Methyloceanibacter*, *Bauldia*, and *Desulfuromonas* were significantly dominant in HS soils (*P* < 0.05). In MS soils, *Bacillus*, *Woeseia*, *Haliangium*, and *PAUC26f* displayed significant dominance (*P* < 0.05). In LS soils, *RB41*, *MND1*, *Nitrospira*, *Gaiella*, *Streptomyces*, *Arthrobacter*, and *Hyphomicrobium* were significantly dominant (*P* < 0.05). Similarly, ten dominant fungal genera exhibited significant differences across sampling sites (Fig. 2D). In HS soils, *Cutaneotrichosporon*, *Emericellopsis*, and *Apiotrichum* were significantly dominant (*P* < 0.05). In MS soils, *Penicillium* and *Rhodotorula* showed significant dominance (*P* < 0.05). In LS soils, *Mortierella*, *Metarhizium*, *Neocosmospora*, *Neonectria*, and *Sarocladium* were significantly dominant (*P* < 0.05).

### Effects of environmental factors on bacterial and fungal communities

**Fig. 3:**
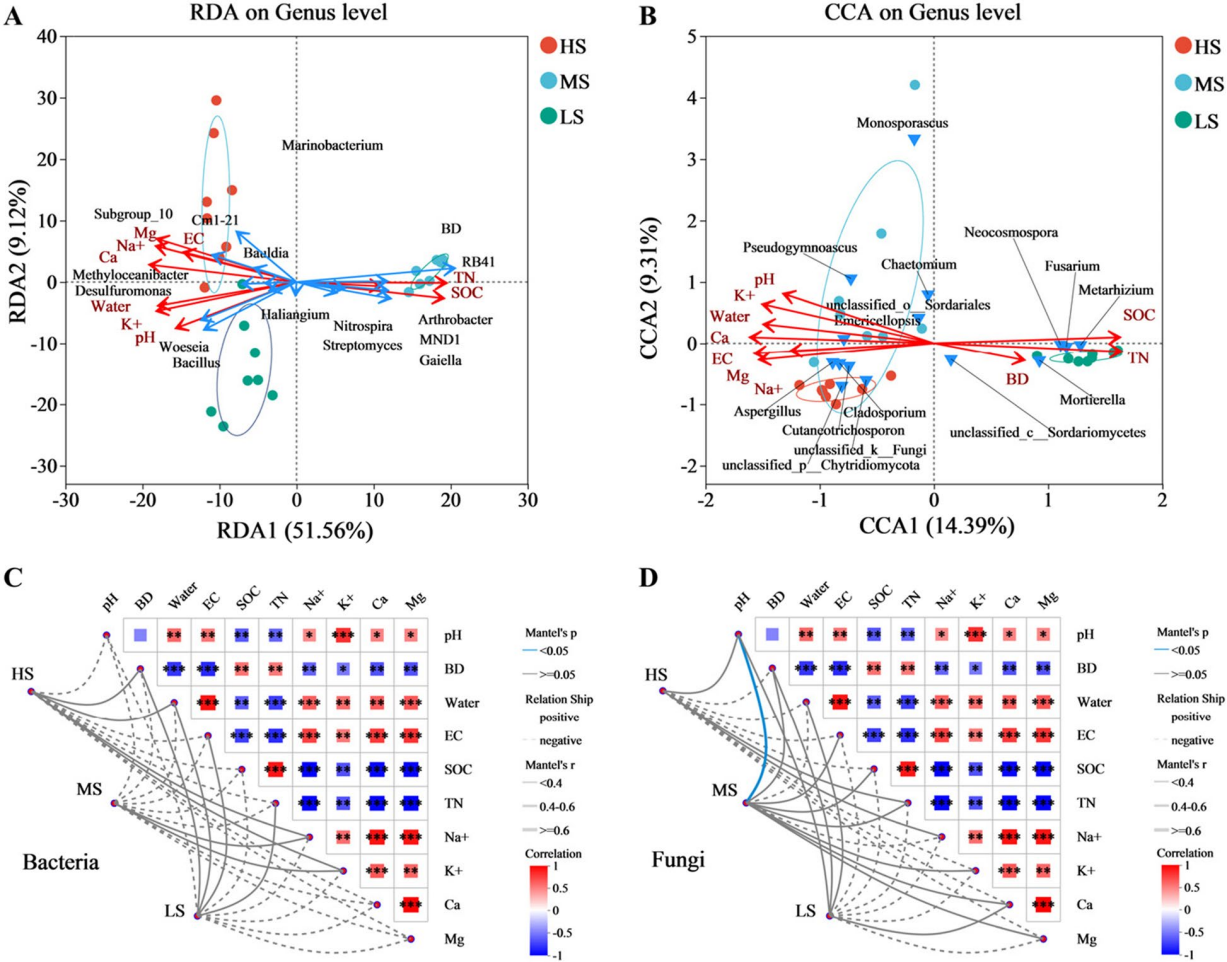
Effects of environmental factors on bacterial (**A**, **C**) and fungal (**B**, **D**) communities in soil. **C**, **D** Line thickness: Indicates the magnitude of the correlation between the community and environmental factors, depicted using the absolute value of Mantel’s r (|R|); Relationship: "Positive" and "Negative" denote the direction (positive or negative) of the correlation between the community and environmental factors; In the heatmap, different colors represent positive or negative correlations, with color intensity indicating the strength of these correlations. Asterisks within the colored blocks denote statistical significance. * 0.01 < *P* ≤ 0.05, ** 0.001 < *P* ≤ 0.01, *** *P* ≤ 0.001

Relationships among samples, environmental factors, and microbial communities (genus level for dominant species) were analyzed. The results demonstrated a clear separation among bacterial communities across the three sites, with significant differences observed (Fig. 3A, Supplementary Fig. 6 A). Fungal communities in HS and MS soils exhibited partial overlap but remained distinctly separated from those in LS soils (Fig. 3B, Supplementary Fig. 6B).

For bacterial communities, SOC, BD, and TN were significantly positively correlated with the community structure in LS soils, especially with *RB41*, *MND1*, *Gaiella*, and *Nitrospira*. In contrast, pH, EC, and ion concentrations (e.g., Na, K, Ca, Mg) were significantly positively correlated with bacterial community structure in HS and MS soils, particularly with *Bacillus*, *Woeseia*, and *Subgroup_10* (Fig. 3A, C).

Similarly, in fungal communities, SOC, BD, and TN were significantly positively correlated with the community structure in LS soils, especially with *Fusarium* and *Mortierella*. Meanwhile, pH, EC, and ion levels of Na, K, Ca, and Mg were significantly positively correlated with fungal community structures in HS and MS soils, particularly with *Aspergillus*, *Emericellopsis*, and *Cutaneotrichosporon* (Fig. 3B, D).

**Fig. 4:**
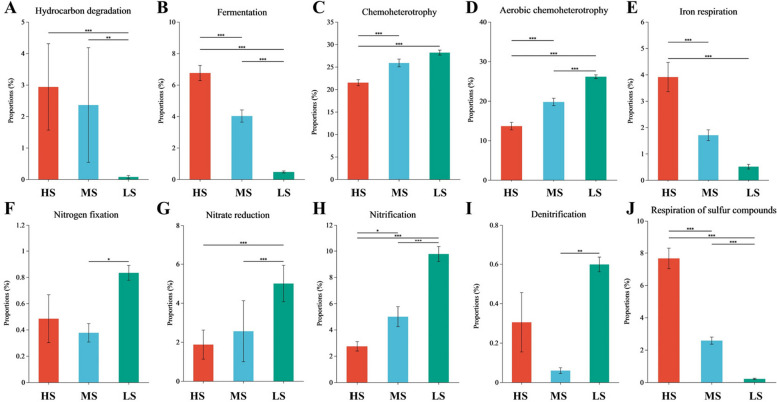
Functional analysis of bacterial communities based on the FAPROTAX database. *P < 0.05; **P < 0.01; ***P < 0.001 (One-way ANOVA)

A comparison of genus-level taxa across samples, along with their corresponding metabolic or ecological functions in the FAPROTAX database, revealed notable trends in bacterial community functionality. The results showed that, with increasing soil salinity, the relative abundance of bacterial communities associated with hydrocarbon degradation, fermentation, iron respiration, and sulfur compound respiration significantly increased (*P* < 0.05) (Fig. 4A, B, E, J). In contrast, bacterial communities related to chemoheterotrophy, aerobic chemoheterotrophy, nitrate reduction, and nitrification exhibited a gradual decline in relative abundance (*P* < 0.05) (Fig. 4C, D, G, H). The relative abundance of nitrogen-fixing and denitrifying bacteria showed no significant difference between MS and HS soils, though both were significantly lower than in LS soils (*P* < 0.05) (Fig. 4F, I).

**Fig. 5:**
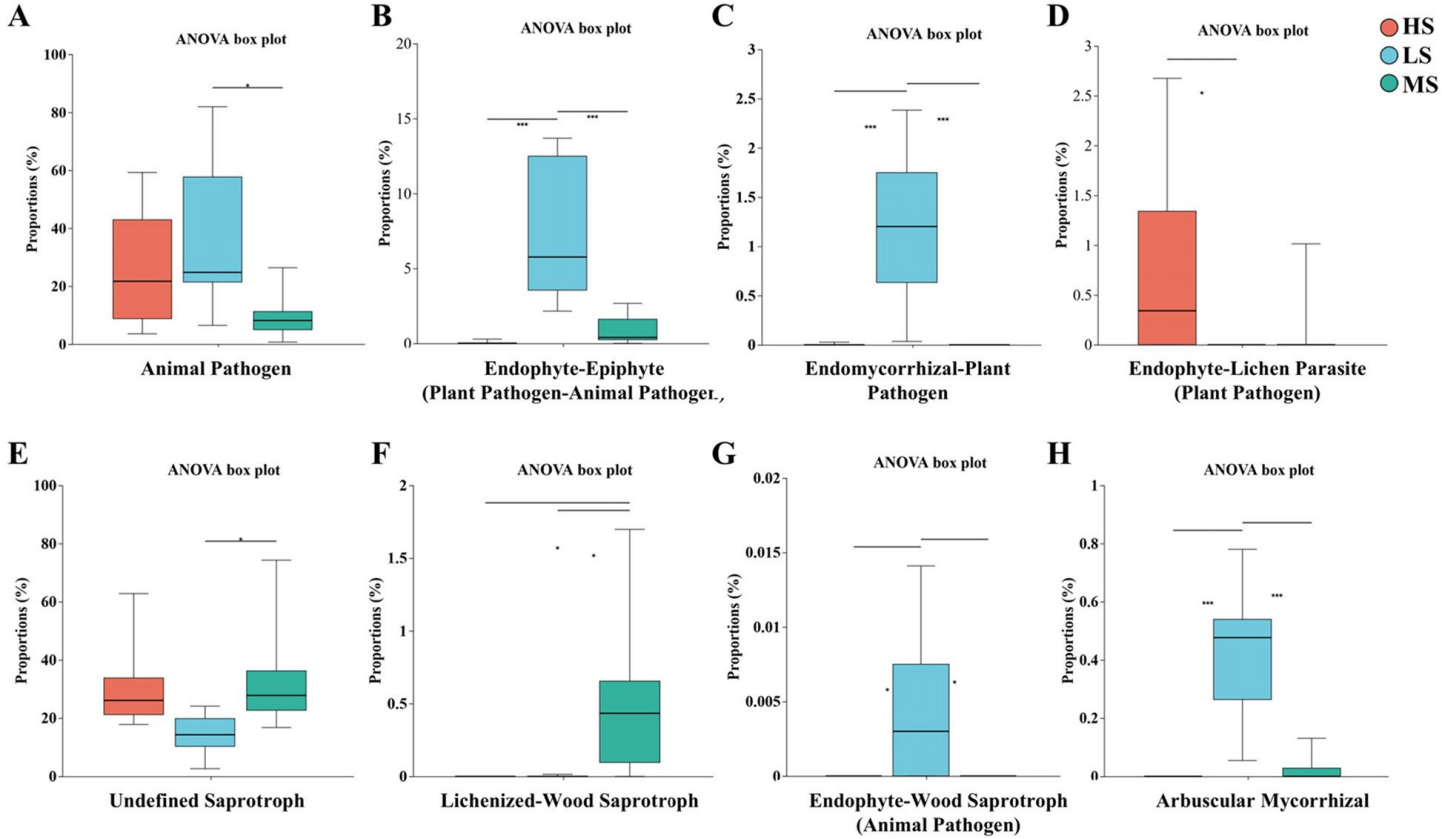
Functional analysis of fungal communities based on FUNGuild. *P < 0.05; **P < 0.01; ***P < 0.001 (One-way ANOVA)

Fungal communities were classified by function and compared for relative abundance across sampling sites using FUNGuild. The results showed distinct functional characteristics among sites. In LS soils, fungi associated with Endophyte-Epiphyte (Plant Pathogen-Animal Pathogen), Endomycorrhizal-Plant Pathogen, Endophyte-Wood Saprotroph (Animal Pathogen), and Arbuscular Mycorrhizal functions had significantly higher relative abundance than in HS and MS soils (*P* < 0.05) (Fig. 5B, C, G, H). In MS soils, Animal Pathogen-associated fungi were significantly less abundant than in LS soils (*P* < 0.05) (Fig. 5A), while fungi with the Undefined Saprotroph function showed significantly higher abundance than in LS soils (*P* < 0.05) (Fig. 5E). In HS soils, fungi with the Endophyte-Lichen Parasite (Plant Pathogen) function were significantly more abundant than in LS soils (*P* < 0.05) (Fig. 5D).

**Fig. 6:**
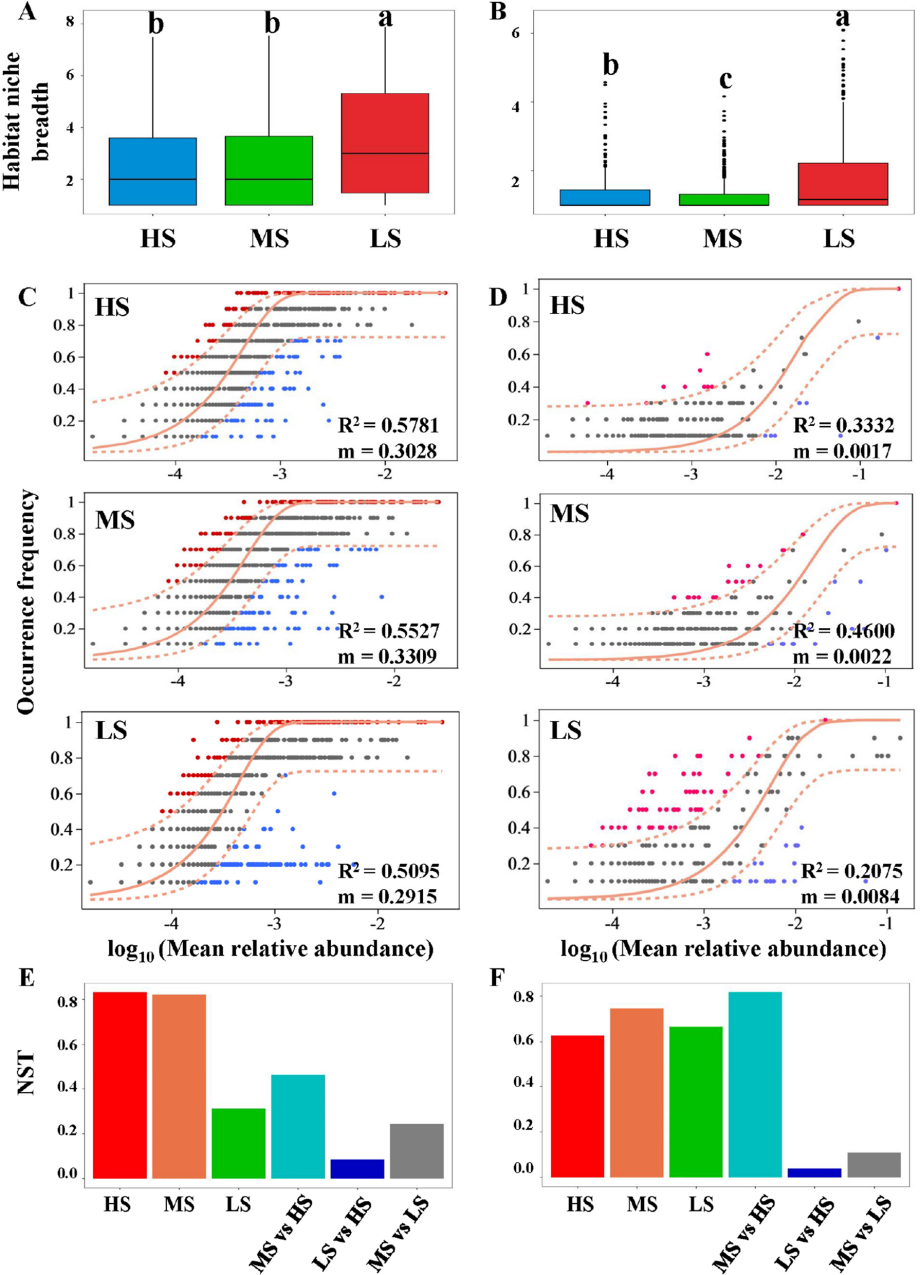
Niche and neutral processes in microbial community assembly in saline-alkali soils. **A**, **B** Habitat niche breadth in different sites for bacterial (**A**) and fungal (**B**) communities. **C**, **D** Neutral models assessing the impact of neutral processes on bacterial (**C**) and fungal (**D**) community assembly, with orange shading showing the 95% confidence interval predicted by the model. R^2^ indicates goodness of fit, and *m* represents species migration rate. A higher R^2^ indicates a better fit to the neutral model, meaning a stronger influence of stochastic processes and a weaker influence of deterministic processes on community assembly. The parameter m quantifies the community-level migration rate. It is assumed to be the same for all taxa in a community (i.e., species-independent). A smaller m indicates stronger dispersal limitation, whereas a larger m indicates weaker dispersal limitation. **E**, **F** NST models evaluating the relative importance of stochastic processes in bacterial (**E**) and fungal (**F**) community assembly. NST values above 0.5 suggest dominance by stochastic processes, while values below 0.5 indicate a predominance of deterministic processes

To evaluate the roles of niche-based (deterministic) and neutral (stochastic) processes in saline-alkaline soil microbial communities, we calculated habitat niche breadth and built neutral models and NST null models. For both bacterial and fungal communities, habitat niche breadth was higher in low-salinity environments than in high-salinity environments (Fig. 6A, B). In addition, the lower R^2^ values of the neutral models for bacterial and fungal communities in LS soils indicate that community assembly in LS was less influenced by stochastic processes and more influenced by deterministic processes (Fig. 6C, D). Differences in the migration rate (m) suggested distinct patterns for bacteria and fungi: in LS soils, bacteria showed stronger dispersal limitation, whereas fungi showed weaker dispersal limitation (Fig. 6C, D). The NST null models further indicated that stochasticity dominated microbial community assembly in high-salinity environments. Notably, bacterial assembly in low-salinity environments was mainly driven by deterministic factors, whereas fungal assembly was mainly driven by stochastic processes (Fig. 6E, F).

**Fig. 7:**
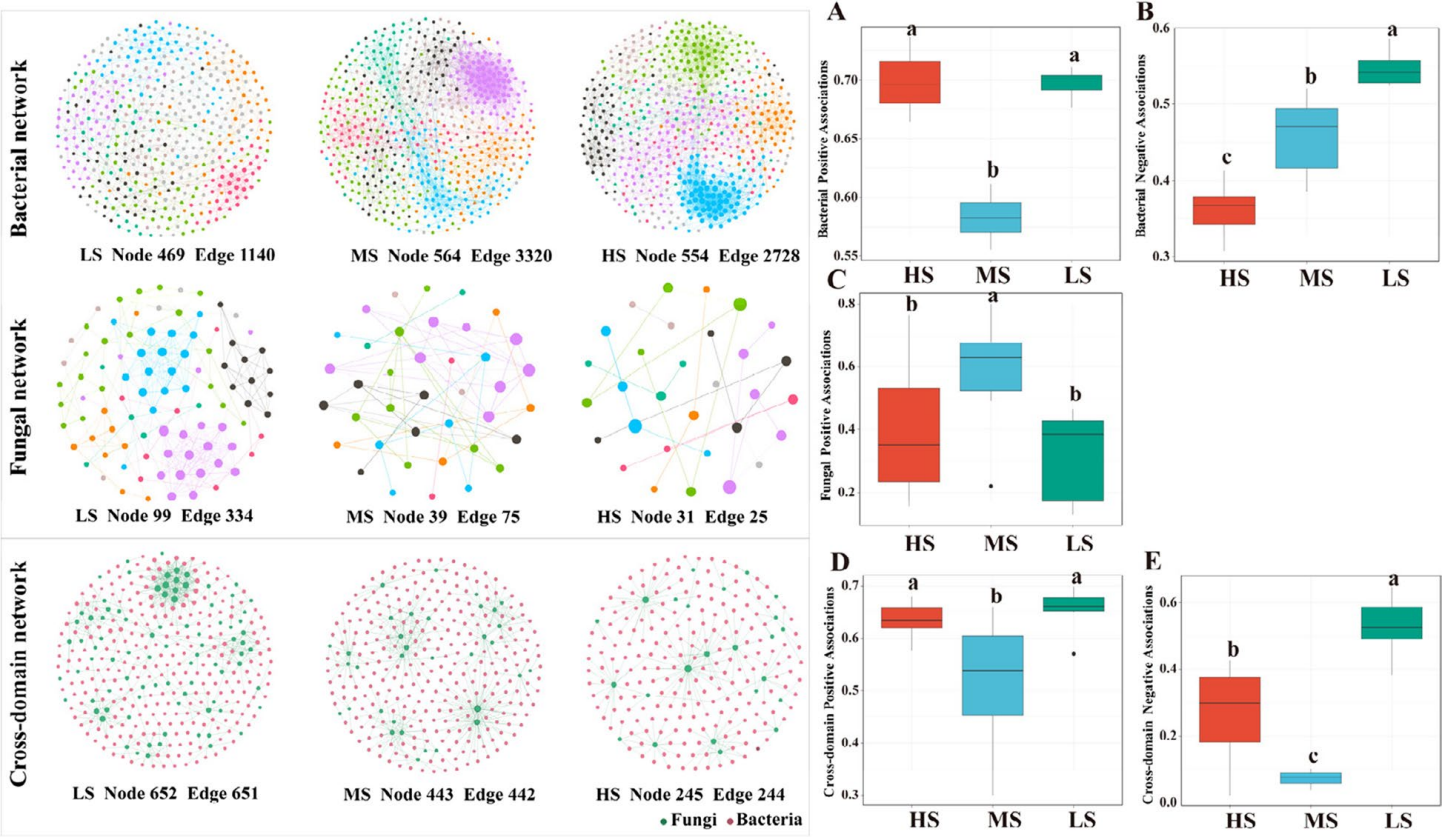
Microbial networks and keystone species in different saline-alkali soil sites

Network analysis revealed that bacterial biological associations differed significantly among treatment groups (Fig. 7). In high-salt environments, bacterial communities exhibited the highest positive biological associations (Fig. 7A) and the lowest negative biological associations (Fig. 7B). In low-salt environments, bacterial communities showed tighter biological associations, particularly with significantly higher negative biological associations compared to medium- and high-salt environments (Fig. 7B). Due to relatively simpler fungal networks, negative biological associations were not detected in fungal communities; however, their positive biological associations still showed significant differences across salt concentration environments (Fig. 7C). Through constructing cross-domain biological association networks between bacteria and fungi, results indicated that in high-salt environments, both domains adopted more active interaction strategies (Fig. 7D). Similarly, in low-salt environments, cross-domain negative associations between bacteria and fungi were significantly higher than in other salt environments (Fig. 7E).

## Discussion

### Impact of salinization on soil microbial community composition and function

Soil microorganisms are essential in decomposing plant residues and soil organic matter, playing a key role in biogeochemical cycles [79]. Consistent with previous studies, we found that different saline-alkali conditions support distinct microbial communities [68, 69], potentially influencing community structure and evolution within these ecosystems [1, 51]. We further analyzed the effects of environmental factors on microbial community composition (Fig. 3). Results showed that while bacterial and fungal communities across sites shared dominant phyla (Proteobacteria and Ascomycota), there were significant differences at the genus level. Soil physicochemical properties universally impact the diversity and structure of bacterial communities [41, 48]. Here, bulk density (BD), total nitrogen (TN), and soil organic carbon (SOC) were positively correlated with bacterial and fungal community structures in LS soils, whereas electrical conductivity (EC), water content, potassium (K), pH, calcium (Ca), magnesium (Mg), and sodium (Na) were positively correlated with microbial communities in HS and MS soils (Fig. 3). *Proteobacteria* primarily use rhizosphere carbon, while *Firmicutes* dominate in straw carbon decomposition [14]. *Ascomycota* is efficient in carbohydrate decomposition but does not break down lignin [17]. Extracellular enzymes convert organic substrates into inorganic and soluble forms, directly affecting SOC dynamics. Compared to the limited mobility of bacteria, fungi provide significant physicochemical protection for SOC [54].

Current research primarily focuses on microbial diversity in saline-alkali soils under various land-use types, whereas studies on microbial function, succession, and migration within naturally succeeding sites are limited [11]. This study examined functional differences in microbial communities across sites with representative vegetation. Within bacterial communities, *Nitrospira* was most abundant in LS soils and decreased as salinity increased (Fig. 2A, C). Bacteria associated with nitrogen fixation, nitrate reduction, nitrification, and denitrification were significantly more abundant in LS soils than in HS and MS soils (Fig. 4F, G, H, I).

Conversely, bacteria involved in hydrocarbon degradation, fermentation, and iron respiration were less abundant in LS soils than in HS and MS soils. This may be due to the high petroleum degradation, organic matter decomposition, and ion transport capabilities of these genera, which thrive in the high-salinity soils near oilfields in HS and MS plots. Fungal richness in LS soils was significantly higher than in HS and MS (*P* < 0.05), yet the relative abundance of fungi with undefined saprotrophic and lichenized-wood saprotrophic functions was lower in high-salinity soils (Fig. 5E, F). These findings suggest that land-use and vegetation changes partially influence soil microbial processes related to nitrogen and carbon cycling [40]. High-salinity soils harbor a richer abundance of saprotrophic bacteria and fungi.

Arbuscular mycorrhizal fungi (AMF), while not “sustainable saviors” for intensive agriculture [60], have potential for aiding crops in nutrient assimilation [61]. The tripartite symbiosis of nitrogen-fixing bacteria, AMF, and crops synergistically enhances crop biomass and nitrogen fixation, with effects that surpass rhizobia-AMF-crop symbiosis [50]. Previous research shows that under stress conditions, AM fungi contribute more to host growth [13]. Although this study could not identify all nitrogen-fixing bacteria and AMF, functional analysis showed significant enrichment of both in LS soils (Figs. 4F, 5H), suggesting a positive interaction. Therefore, nitrogen-fixing bacteria and AMF from low-salinity soils may offer synergistic benefits for sustainable forestry and agriculture in saline-alkaline environments. In contrast, HS and MS soils had lower levels of AMF, suggesting high-salinity soils may be unsuitable for highly active AMF.

Pathogen-associated fungi were also notably abundant across sites. Plant and animal pathogens had relatively high proportions in LS soils (Fig. 5A, B, C). This may be due to soil physicochemical properties. Soil microorganisms predominantly drive nutrient supply and transport in terrestrial ecosystems [59]. Increased microbial activity and richness enhance nutrient release, benefiting pathogens, which are strong competitors for nutrients [4]. CCA analysis confirmed positive correlations between TN, BD, and SOC with *Fusarium* (Fig. 3B). Additionally, LS forests support large populations of insects and undergrowth, providing carbon and energy sources for microbial growth via feces and remains [33, 34]. Although fungal richness varied significantly across sites, fungal diversity showed no significant difference, suggesting functional redundancy within the fungal community that helps maintain stability in community diversity [31].

### Mechanisms controlling the assembly of microbial communities in saline-alkali soils under different levels of salinity

Soil microorganisms influence soil health by regulating organic matter decomposition, nutrient cycling, and energy flow. The life history strategies of microorganisms determine how they allocate limited metabolic resources among growth, resource acquisition, and stress tolerance [32]. This study examined the role of neutral (stochastic) processes in structuring microbial communities within naturally succeeding environments. First, microbial communities in LS soils were found to be less affected by environmental factors. Although bacterial communities showed greater dispersal limitations, fungal communities displayed higher migration rates. This trend aligns with variations in SOC content (Fig. 6). This effect may stem from high habitat heterogeneity and a broader niche breadth in LS soils [12, 15, 29]. Specifically, in areas with high vegetation cover, the dispersal of pathogenic and arbuscular mycorrhizal fungi reduces dispersal limitations for fungi, thereby enhancing the influence of neutral processes in community assembly [30, 36, 67]. Based on the microbial co-occurrence network, we constructed a more robust microbial biological association network. This network addresses limitations inherent in correlation matrix-based co-occurrence networks, such as spurious associations, autocorrelation, and correlation spillover. It further excludes co-occurrence signals arising from environmental selection or niche overlap, ensuring that associations within the microbial biological association network predominantly represent signals generated by microbial life activities. Our findings reveal that in high-salt environments, bacterial communities exhibit the highest positive biological associations (Fig. 7A) and the lowest negative biological associations (Fig. 7B). This pattern suggests that under more severe environmental stress, microbial communities may adopt mutualistic symbiosis as a survival strategy. According to microbial life-history strategy theory, under environmental stress, microbial communities often employ the S-strategy to better withstand stressors [38, 39, 62, 71]. However, this adaptation involves trade-offs, including reduced resource acquisition capabilities. Consequently, such microorganisms may require greater collaborative mutual assistance from other community members. Based on the Black Queen Hypothesis, approximately 20–50% of microorganisms actively lose redundant metabolic pathways (e.g., amino acid and nucleotide synthesis) under resource limitation and environmental stress, evolving into obligate auxotrophs [26, 43]. This forces communities to form obligate reciprocal division of labor networks—where auxotrophs exchange essential nutrients by secreting private metabolites [18]. This aligns with our observed phenomenon (Fig. 7A). Additionally, in low-salt environments, bacterial communities exhibit tighter biological associations, particularly with significantly higher negative biological associations compared to MS and HS environments (Fig. 7B). This represents a more stable network state. Generally, networks with higher negative associations are more stable [22, 73], as under fluctuating environmental conditions, positive associations within microbial communities may shift synchronously, causing community-wide resonance fluctuations, while negative associations reduce such resonance and enhance stability [7, 9]. Due to relatively simpler fungal networks, we detected no negative biological associations in fungal communities; however, their positive biological associations still showed significant differences across salt concentration environments (Fig. 7C). We also constructed cross-domain biological association networks between bacteria and fungi. Similar to intra-bacterial networks, in HS environments, bacteria and fungi adopted more active interaction strategies (Fig. 7D). Conversely, in LS environments, cross-domain negative associations between bacteria and fungi were significantly higher than in other salt environments (Fig. 7E). Of course, these conclusions still require genome-level validation for full confirmation.

Salt stress affects soil nutrients, which may favor well-adapted taxa, enhancing their competitive edge [21, 37]. Results showed that SOC drive shifts in microbial community composition (Supplementary Fig. 6). This pattern likely emerges when microorganisms, particularly saprotrophic fungi, compete for these resources, leading to intense interactions within taxa due to limited niche space [19]. Such interspecies competition shapes specific community composition and functions centered on SOC as key environmental factors, supporting functional divergence among fungi across sites (Fig. 3). Additionally, the average clustering of nodes in the LS network decreased, while nitrogen-cycling microbes increased (Fig. 4), suggesting that closer coupling among key nodes enhances communication within the network. A moderate reduction in bacterial diversity may improve nitrogen metabolic efficiency. Stability in soil microbial ecosystems is associated with function-specific keystone microbes [52]. Thus, changes in key nodes may impact network stability. In this study, certain species were identified as key taxa related to microbial community stability under salt rd, mainly from *Proteobacteria*, *Chloroflexi*, *Ascomycota*, and *Basidiomycota* (Supplementary Fig. 6). Among them, *Bacillus* exhibited high salt tolerance and was involved in nitrogen metabolism [58], showing an increase in relative abundance in higher-salinity soils (Fig. 2A, C). However, this did not increase nitrogen-fixing activities, likely due to the wide distribution of this keystone group in varied ecosystems like wetlands, forests, and farmlands [28]. Thus, shifts in key taxa abundance due to environmental disturbance may negatively impact microbial stability. On the other hand, animal and plant pathogen abundance decreased in higher-salinity soils (Fig. 5A, B, C, G), possibly due to the antimicrobial effects of many *Bacillus* species. Thus, research on microbial resources and functional connections across diverse habitats could provide valuable insights for designing microbial communities with targeted functions.

Understanding the fundamental mechanisms that drive microbial community assembly and species coexistence is central to ecology and crucial for connecting microbial community stability with ecosystem function. Community assembly is often explained through deterministic and stochastic processes [44, 78]. Deterministic processes involve ecological selection, where species presence and abundance are shaped by predictable factors, including biotic factors (e.g., intra-species competition and predation) and abiotic factors (e.g., environmental conditions like pH and temperature) [63, 76]. Stochastic processes, in contrast, involve unpredictable disturbances, random dispersal, and birth–death events affecting species turnover [3, 10]. Identifying mechanisms that balance stochastic and deterministic processes under environmental shifts can help clarify community assembly [25]. For example, high salinity often drives deterministic assembly of microeukaryotic plankton communities [42]. Similarly, climate warming may influence soil bacterial assembly, showing strong correlations with drought and precipitation [47]. However, the impact of environmental factors—especially amino sugar variations—on bacterial and fungal community assembly in naturally succeeding, salt-stressed environments without human interference remains uncertain.

As geographical distance between samples increases, microbial community similarity declines significantly, indicating an uneven spatial distribution and suggesting a distance-decay relationship among microbial communities within this niche. Larger differences in environmental factors correspond to lower microbial community similarity, emphasizing the role of environmental conditions in shaping community structure.

### Study limitations

Although space-for-time substitution is a classic approach, we cannot completely rule out the influence of unmeasured historical factors among sites (e.g., microtopographic differences). In addition, amplicon-based functional prediction (FAPROTAX/FUNGuild) only reflects metabolic potential and cannot replace evidence of activity from metatranscriptomics or proteomics. Future work should combine long-term field monitoring with multi-omics approaches to validate the assembly model proposed here.

## Conclusion

A key challenge in studying the effects of salinity on soil microbial communities lies in distinguishing salinity impacts from those of other potentially correlated variables. This study, conducted at long-term undisturbed sampling sites, investigated the responses of soil microbial composition and function to environmental factors and analyzed the functional characteristics of microbial communities under different conditions. Results revealed that as salinity increased, bacterial richness increased significantly, while fungal richness and bacterial diversity decreased markedly. Community composition and function were strongly filtered by salinity and significantly influenced by soil organic carbon (SOC) content. In low-salinity soils, SOC accumulation promoted the enrichment of nitrogen-fixing bacteria and arbuscular mycorrhizal fungi. In higher-salinity soils, stochastic processes dominated community assembly, with bacteria exhibiting the highest positive associations and lowest negative associations; in low-salinity soils, deterministic processes prevailed, with bacterial negative associations significantly higher than in moderate-to-high salt environments. Cross-domain network analysis showed more active bacterial-fungal interactions in high-salt environments. This study not only deepens understanding of microbial community assembly mechanisms in saline ecosystems but also provides a scientific basis for the effective development and sustainable management of saline soils by elucidating the synergistic regulatory patterns of salinity and SOC, clarifying microbial functional differentiation characteristics, and establishing cross-domain interaction pattern maps.

## Supplementary Information

Supplementary Material 1: Supplementary Figure 1 Rarefaction Curve and Shannon curve. (A) bacterial dilution curves; (B) the shannon-wiener curve of bacteria; (C) fungal dilution curves; (D) the shannon-wiener curve. Supplementary Figure 2 Soil bacterial and fungal α-diversity differs among salinity sites. Different letters denote significant differences among sites (one-way ANOVA, P < 0.05). Supplementary Figure 3 Venn diagram constructed at the microbial OTUs level. (A) the number of bacteria OTUs that are not equally and common, and (B) the number of fungus OTUs that are not equally endemic and common. Supplementary Figure 4 The characteristics of microbial composition in different samples were analyzed using ternary analysis. (A) Bacteria; (B) Fungi. The three vertices of the diagram correspond to three distinct samples. Solid circles represent species identified at the genus level, with circle size proportional to their average relative abundance. Supplementary Figure 5 Relative abundance of microbial in each sample at the genus level. (A) Bacteria; (B) Fungi. The horizontal axis represents the sample names, while the vertical axis indicates the proportion of each species within its respective sample. Bars of different colors represent distinct species, and the length of each bar reflects the proportion of that species. Supplementary Figure 6 Bipartite correlation network analysis illustrating interactions between taxa and environmental factors. (A) Bacteria; (B) Fungi. The top 25 most abundant taxa at the genus level were identified based on Spearman rank correlation coefficients with significant environmental factors (P < 0.05). Node size represents species abundance, with different colors indicating distinct species. Red edges indicate positive correlations, while green edges indicate negative correlations. The thickness of the edges reflects the strength of the correlation coefficient: thicker edges denote stronger correlations. A higher number of edges signifies denser connections between the nodes.

Supplementary Material 2: Supplementary Table 1 Diversity and richness indices of bacterial and fungal communities

Supplementary Material 3. Supplementary Table 2 The niche breadth of bacteria in different plots

Supplementary Material 4. Supplementary Table 3 The niche breadth of fungal in different plots

## Data Availability

The data that support the findings of this study are openly available in National Center for Biotechnology Information at https://www.ncbi.nlm.nih.gov, reference number PRJNA1206298 and PRJNA1206731. https://www.ncbi.nlm.nih.gov/bioproject/PRJNA1206298/. https://www.ncbi.nlm.nih.gov/bioproject/PRJNA1206731/.
