# Supplementary material for: Properties and ecological assembly mechanisms of microbial communities across salinity levels in coastal saline-alkaline soils
Source: BMC Plant Biol. 2026 Mar 20;26:768. doi: 10.1186/s12870-026-08391-3 (PMC13126930; doi:10.1186/s12870-026-08391-3)
Supplement: Supplementary file 1 — Supplementary Material 1: Supplementary Figure 1 Rarefaction Curve and Shannon curve. (A) bacterial dilution curves; (B) the shannon-wiener curve of bacteria; (C) fungal dilution curves; (D) the shannon-wiener curve. Supplementary Figure 2 Soil bacterial and fungal α-diversity differs among salinity sites. Different letters denote significant differences among sites (one-way ANOVA, P < 0.05). Supplementary Figure 3 Venn diagram constructed at the microbial OTUs level. (A) the number of bacteria OTUs that are not equally and common, and (B) the number of fungus OTUs that are not equally endemic and common. Supplementary Figure 4 The characteristics of microbial composition in different samples were analyzed using ternary analysis. (A) Bacteria; (B) Fungi. The three vertices of the diagram correspond to three distinct samples. Solid circles represent species identified at the genus level, with circle size proportional to their average relative abundance. Supplementary Figure 5 Relative abundance of microbial in each sample at the genus level. (A) Bacteria; (B) Fungi. The horizontal axis represents the sample names, while the vertical axis indicates the proportion of each species within its respective sample. Bars of different colors represent distinct species, and the length of each bar reflects the proportion of that species. Supplementary Figure 6 Bipartite correlation network analysis illustrating interactions between taxa and environmental factors. (A) Bacteria; (B) Fungi. The top 25 most abundant taxa at the genus level were identified based on Spearman rank correlation coefficients with significant environmental factors (P < 0.05). Node size represents species abundance, with different colors indicating distinct species. Red edges indicate positive correlations, while green edges indicate negative correlations. The thickness of the edges reflects the strength of the correlation coefficient: thicker edges denote stronger correlations. A higher number of edges sig [file 12870_2026_8391_MOESM1_ESM.pdf]

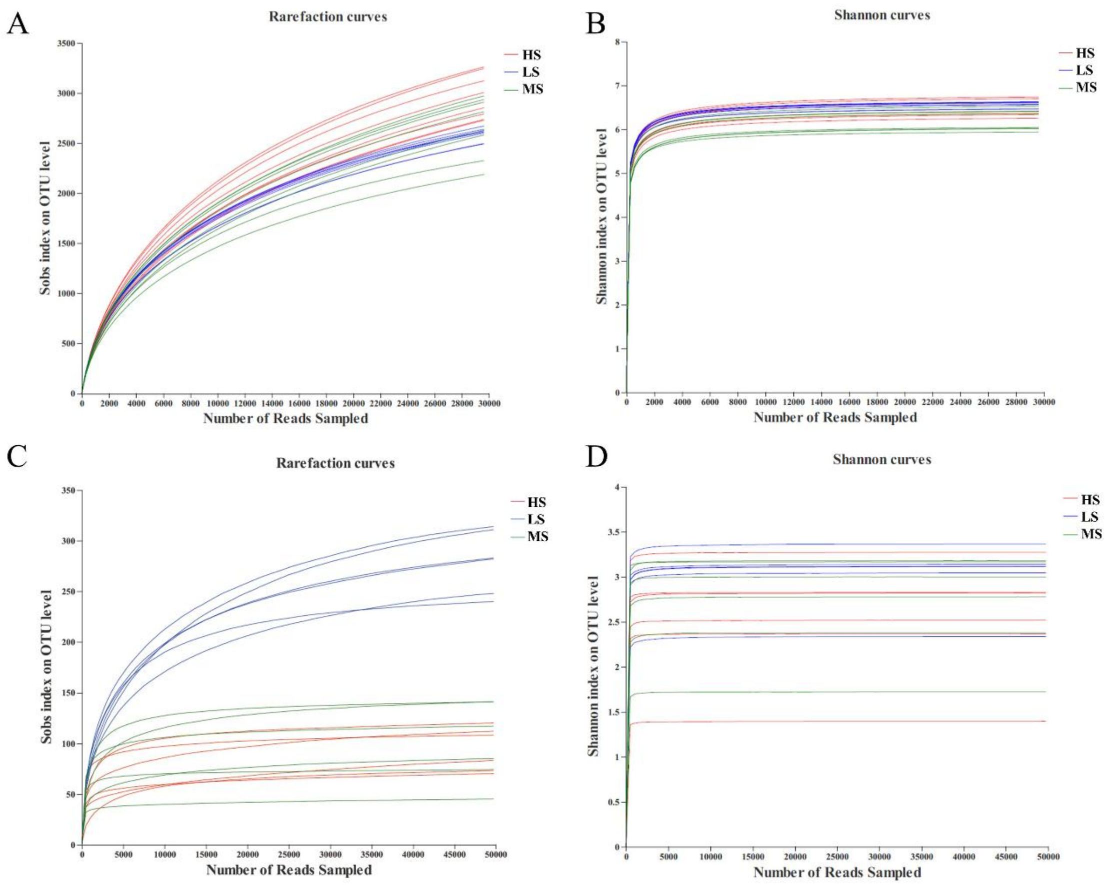

Supplementary Figure 1 Rarefaction curve and Shannon curve. (A) bacterial dilution curves; (B) the shannon-wiener curve of bacteria; (C) fungal dilution curves; (D) the shannon-wiener curve.

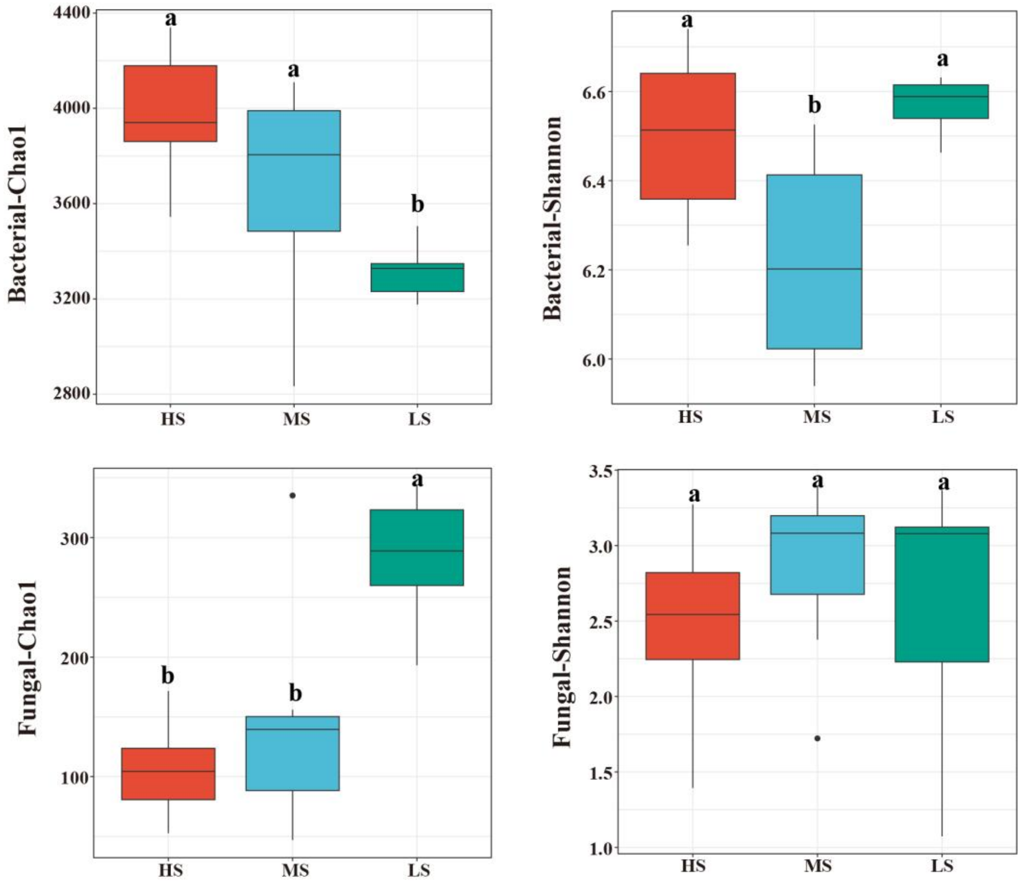

Supplementary Figure 2 Soil bacterial and fungal α-diversity differs among salinity sites. Different letters denote significant differences among sites (one-way ANOVA, $P < 0.05$).

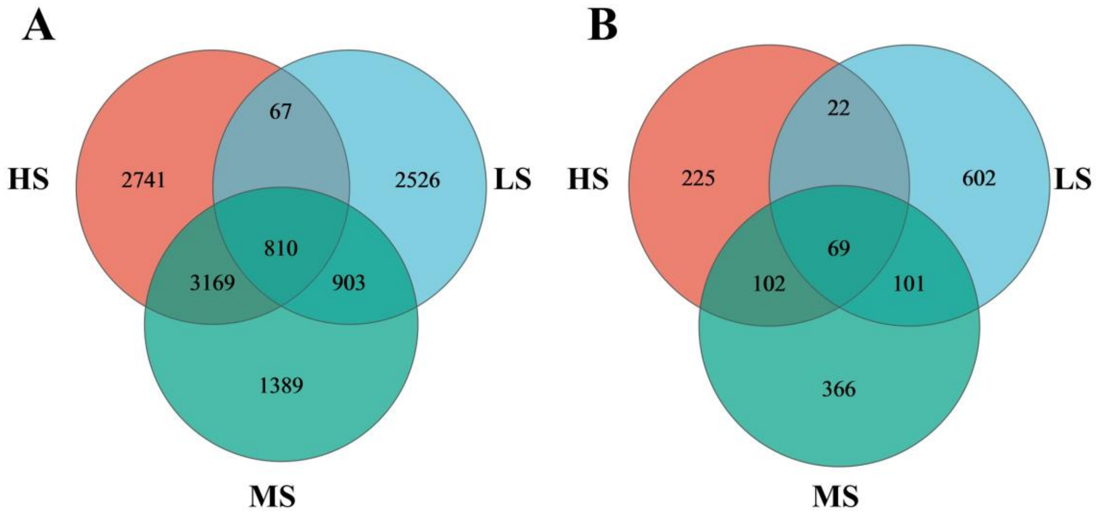

Supplementary Figure 3 Venn diagram constructed at the microbial OTUs level. (A) the number of bacteria OTUs that are not equally and common, and (B) the number of fungus OTUs that are not equally endemic and common.

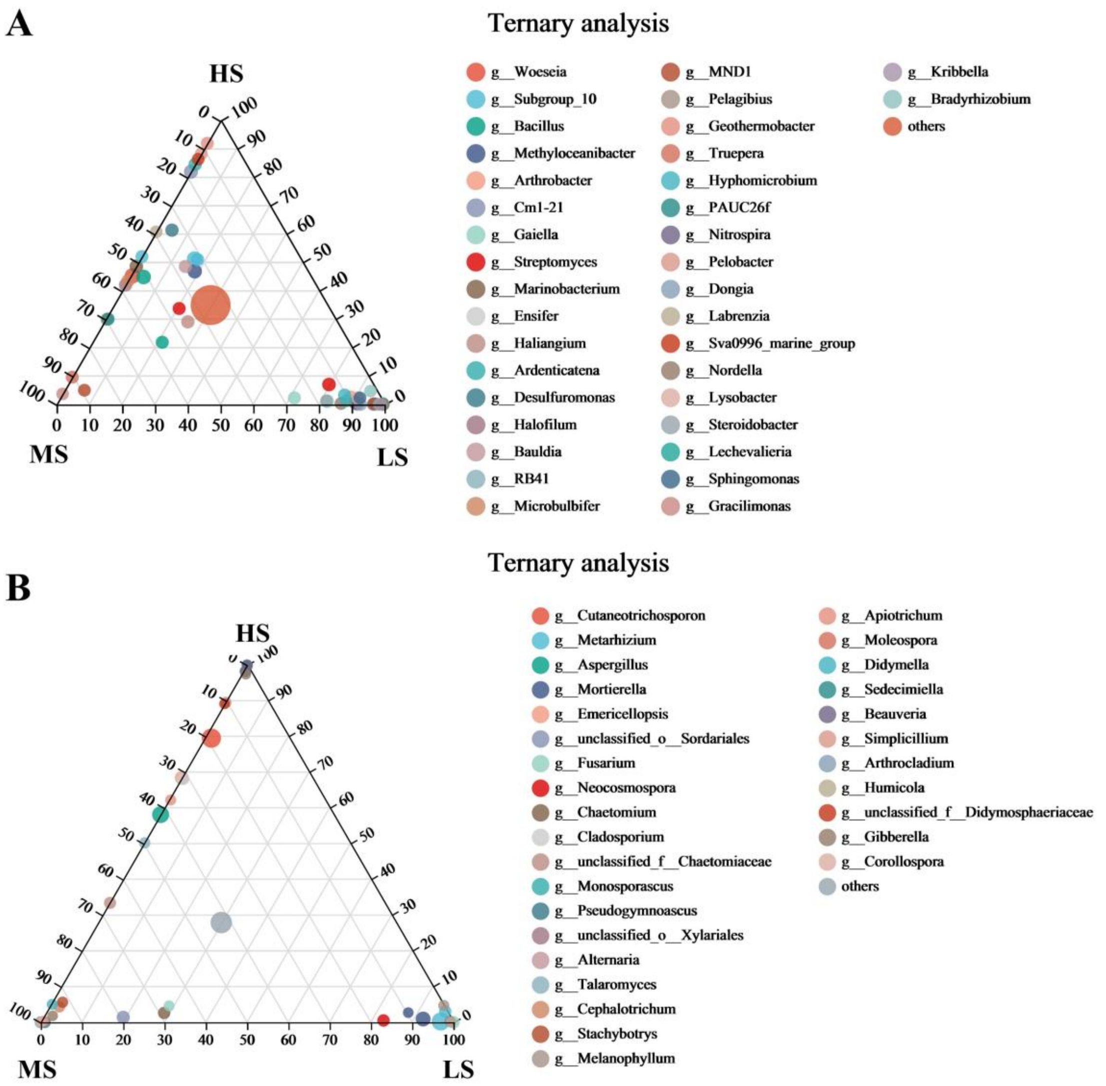

Supplementary Figure 4 The characteristics of microbial composition in different samples were analyzed using ternary analysis. (A) Bacteria; (B) Fungi. The three vertices of the diagram correspond to three distinct samples. Solid circles represent species identified at the genus level, with circle size proportional to their average relative abundance.

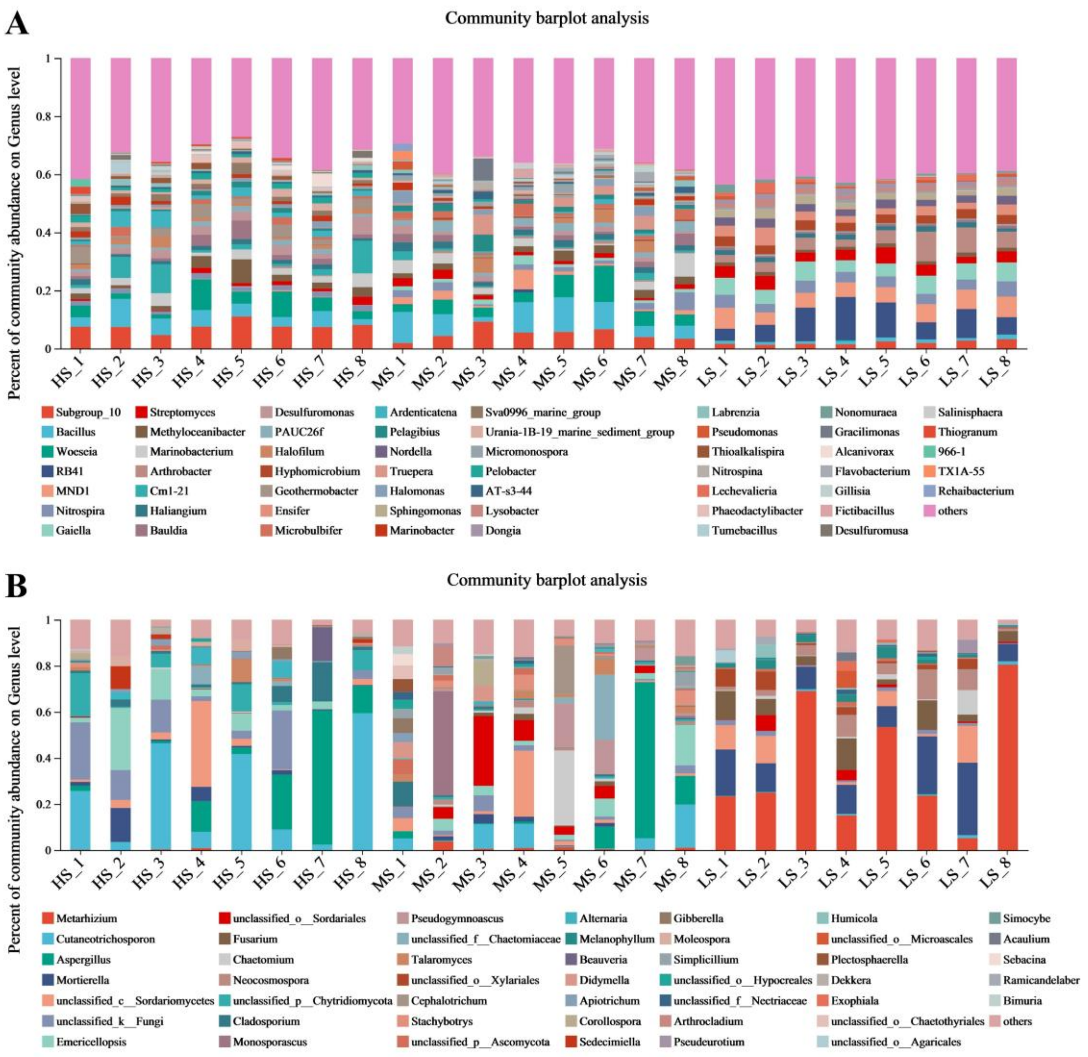

Supplementary Figure 5 Relative abundance of microbial in each sample at the genus level. (A) Bacteria; (B) Fungi. The horizontal axis represents the sample names, while the vertical axis indicates the proportion of each species within its respective sample. Bars of different colors represent distinct species, and the length of each bar reflects the proportion of that species.

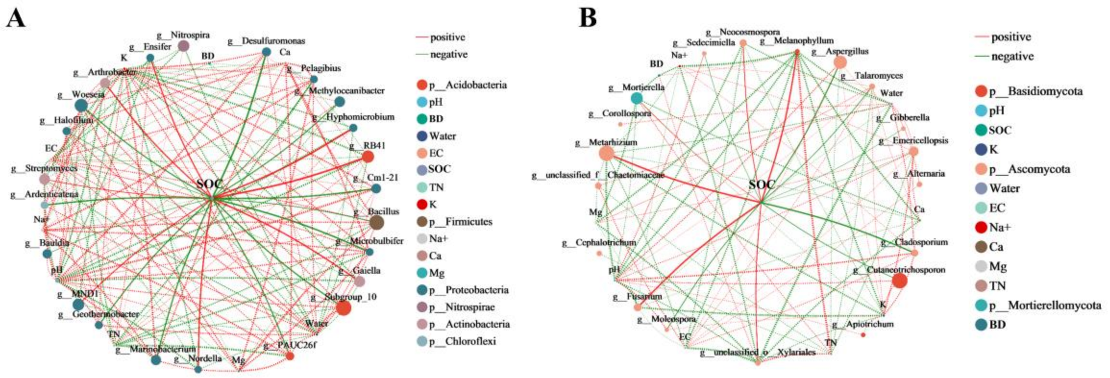

Supplementary Figure 6 Bipartite correlation network analysis illustrating interactions between taxa and environmental factors. (A) Bacteria; (B) Fungi. The top 25 most abundant taxa at the genus level were identified based on Spearman rank correlation coefficients with significant environmental factors ($P < 0.05$). Node size represents species abundance, with different colors indicating distinct species. Red edges indicate positive correlations, while green edges indicate negative correlations. The thickness of the edges reflects the strength of the correlation coefficient: thicker edges denote stronger correlations. A higher number of edges signifies denser connections between the nodes.
