# Supplementary material for: Properties and ecological assembly mechanisms of microbial communities across salinity levels in coastal saline-alkaline soils
Source: BMC Plant Biol. 2026 Mar 20;26:768. doi: 10.1186/s12870-026-08391-3 (PMC13126930; doi:10.1186/s12870-026-08391-3)
Supplement: Supplementary file 2 — Supplementary Material 2: Supplementary Table 1 Diversity and richness indices of bacterial and fungal communities [file 12870_2026_8391_MOESM2_ESM.docx]

Supplementary Table 1 One-way ANOVA of soil bacterial and fungal α-diversity among salinity sites

|  | bacteria | | fungi | |
| --- | --- | --- | --- | --- |
|  | F | P | F | P |
| Chao1 | 7.885 | 0.002 | 18.74 | <0.001 |
| Shannon | 9.058 | 0.001 | 0.751 | 0.484 |
